# The opioid‐impaired provider: A call for national guidance to maximize rehabilitation while protecting patient safety

**DOI:** 10.1002/hsr2.193

**Published:** 2020-10-06

**Authors:** Gretchen LeFever Watson, Nancy J. Selfridge, Bruce E. Wright

**Affiliations:** ^1^ President Safety & Leadership Solutions Norfolk Virginia; ^2^ Departments of Clinical and Medical Foundations Ross University School of Medicine Bridgetown Barbados

## 1 INTRODUCTION

Opioid use disorder (OUD), which includes opioid abuse and addiction, has been at epidemic levels for over a decade. According to the Centers for Disease Control and Prevention (CDC), “In 2017, more than 70,000 people died from drug overdoses, making it a leading cause of injury‐related death in the United States. Of those deaths, almost 68% involved a prescription or illicit opioid.”^1^

Many organizations, including the CDC, the National Institute on Drug Abuse (NIDA), the Department of Health and Human Services (HHS), the Food and Drug Administration (FDA), the American Medical Association (AMA), and the American Dental Association (ADA), have generated guidelines to help reverse the course of this epidemic. Despite providers who abuse or are addicted to opioids (OIPs) being a contributing factor in this multifaceted and enduring epidemic, none of the guidelines specifically address OIP behavior.

Meanwhile, the HHS Secretary recently joined with the Attorney General of the United States Department of Justice (DOJ) to announce expansion of DOJ's Opioid Strike Task Force whose mission is to target and permanently remove from practice providers who abuse their prescription authority.^2^ In the absence of explicit guidance about fostering rehabilitation of OIPs, HHS' recent cooperation with the DOJ is noteworthy.

## 2 A CRITICAL ETHICAL DIVIDE

Unlike national opioid guidelines, both the ADA and the AMA have explicit ethical standards regarding doctors' responsibility to protect impaired providers (exact verbiage contained in Table 1).^3^, ^4^ Similarly, the American College of Physicians Ethics Manual includes: “Every physician is responsible for protecting patients from an impaired physician and for assisting an impaired colleague. Fear of mistake, embarrassment, or possible litigation should not deter or delay identification of an impaired colleague.”^5^ Its related position paper states, “The physician should be rehabilitated and reintegrated into medical practice whenever possible without compromising patient safety.”^6^

**Table 1:** Protection obligations—verbatim extractions from ethical codes

	AMA^a^	ADA^b^
Protect patients	Physicians must recognize responsibility to patients first and foremost	The ADA calls on dentists to follow high ethical standards which have the benefit of the patient as their primary goal
Act on behalf of impaired colleagues	Physicians who are impaired are deserving of thoughtful, compassionate care. Physicians are ethically obligated to: (a) Intervene in a timely manner to ensure that impaired colleagues cease practicing and receive appropriate assistance from a physician health program. (b) Report impaired colleagues in keeping with ethics guidance and applicable law. (c) Assist recovered colleagues when they resume patient care. (d) Collectively, physicians have an obligation to ensure that their colleagues are able to provide safe and effective care	All dentists have an ethical obligation to urge chemically impaired colleagues to seek treatment. Dentists with first‐hand knowledge that a colleague is practicing dentistry when so impaired have an ethical responsibility to report such evidence to the professional assistance committee of a dental society. Anyone who believes that a member‐dentist has acted unethically should bring the matter to the attention of the appropriate constituent (state) or component (local) dental society. Whenever possible, problems involving questions of ethics should be resolved at the state or local level
Protect society	The medical professional should safeguard the public and itself against physicians deficient in moral character or professional competence. Physicians should observe all laws, uphold the dignity and honor of the profession and accept its self‐imposed disciplines. They should expose, without hesitation, illegal, or unethical conduct of fellow members of the profession	The dental profession holds a special position of trust within society. As a consequence, society affords the profession certain privileges that are not available to members of the public‐at‐large. In return, the profession makes a commitment to society that its members will adhere to high ethical standards of conduct
Maintain personal health and wellness	When physician health or wellness is compromised, so may the safety and effectiveness of the medical care provided. To preserve the quality of their performance, physicians have a responsibility to maintain their health and wellness, broadly construed as preventing or treating acute or chronic diseases, including mental illness, disabilities, and occupational stress. Seeking appropriate help as needed, including help in addressing substance abuse. Physicians should not practice if their ability to do so safely is impaired by use of a controlled substance, alcohol, other chemical agent, or a health condition	It is unethical for a dentist to practice while abusing controlled substances, alcohol or other chemical agents which impair the ability to practice
Self‐regulate	Society permits medicine to set standards of ethical and professional conduct for physicians. In return, medicine is expected to hold physicians accountable for meeting those standards and to address lapses in professional conduct when they occur. Collectively, physicians have an obligation to ensure that colleagues are able to provide safe and effective care, which includes promoting health and wellness among physicians	Every profession owes society the responsibility to regulate itself. Such regulation is achieved largely through the influence of the professional societies. All dentists, therefore, have the dual obligation of making themselves a part of a professional society and of observing its rules of ethics

Abbreviations: ADA, American Dental Association; AMA, American Medical Association.

a AMA code of ethics.^3^

b Principles of ethics and code of conduct.^4^

In contrast, the United States Supreme Court has repeatedly affirmed that police and other law enforcement professionals and government employees, including Drug Enforcement Administration (DEA) and DOJ employees, have no constitutional duty to protect citizens unless they are in custody.^7^ When the directive of an agency is to gather enough evidence to obtain a conviction, sometimes law enforcement professionals find it necessary to knowingly keep citizens, including patients and impaired providers, at risk of medical harm.

An example of the contrasting ethos of protecting patients and providers from harm vs punishing providers who harm patients is the case of Gary Hartman. An endodontist by trade, Dr Hartman's practice in Virginia Beach was quite successful, earning him up to $500 000 USD per year.^8^ This ended on October 2, 2019, when Hartman was sentenced to 8 years 4 months in prison for a conspiracy to distribute opioids. This conviction was based on a four‐year investigation by the DEA. Per court and board documents, from 2014 to 2018, Hartman performed surgeries and other invasive procedures under the influence of mind‐altering drugs, including up to 15 painkiller and 3 stimulant pills per day. He wrote over 1000 opioid prescriptions for nonmedical purposes for patients he never saw, illegally dispensing more than 75 000 pills into his community. Hartman also conspired with patients, including those for whom he exchanged free dental services, for opioid prescriptions which they would fill for his personal use.^9^, ^10^

It is hardly conceivable that everyone within Hartman's local healthcare community was unaware of what was going on. Halfway through the investigation, in 2016, the DEA served a search warrant on Hartman at his office, finding marijuana and drug paraphernalia. Hartman also tested positive for opioids, stimulants, and marijuana. He was neither arrested nor was charges filed. His practice continued, and his local medical community did nothing. In 2017, during the same year a pharmacist anonymously reported him to the DEA, one of his patients died from a drug overdose.^11^ Again, the local medical community failed to intervene on behalf of Hartman. By the time the Virginia Board of Dentistry learned about this case in 2018, Hartman had already confessed to the DEA about a chronic alcohol and drug addiction spanning over a decade. Only then could this board take action. By then its only option was to revoke his license. The next year, when Hartman was formally indicted, the court's representative stated that the Hartman case should “stand as a warning to other medical professionals” and “we will not cease our efforts in bringing these types of pill‐pushers to justice.”^9^ A situation should never again progress to the point that a pharmacist must contact law enforcement in the hope of stopping an impaired provider from putting patients at risk.

### 2.1 Opioid abuse and diversion among healthcare providers

A 2019 literature review indicates that substance use disorders affect approximately 8% to 15% of American healthcare professionals, a rate that is on par with the general public.^12^ This means in the United States alone, 1.3 to 2.3 million healthcare professionals are either abusing and/or addicted to drugs and/or alcohol. Doctors and other providers with prescription authority face unique addiction risks because they have easy access to addictive drugs, tend to work under chronically stressful conditions, and personally use opioids at a rate five to eight times higher than the lay public.^13^, ^14^, ^15^ This is not a new concern. Among physicians hospitalized between 1986 and 1991 due to current substance‐related impairment, opioid addiction was diagnosed in 36% of cases.^16^ Because the opioid epidemic exploded only after this study was completed, OUD may now comprise a greater proportion of substance‐related impairment cases than ever before.

According to figures partially generated prior to the explosion of the opioid epidemic, at a minimum, nearly half a million to over one million healthcare professionals suffer from OUD. This estimate, which suggests OUD exists among 2.8% to 5.4% of healthcare providers, seems reasonable given the current rate of OUD among the general public. Namely, according to data from 2012 to 2013 National Epidemiologic Survey on Alcohol and Related Conditions^17^ and as reported in a National Institutes of Health (NIH) press release noting a doubling of nonmedical use of opioids from 2002 to 2013,^18^ 4.1% of United States adults (10 million Americans) suffer from OUD. An undocumented, though not irrelevant, number of these 10 million OUD sufferers are healthcare providers. If even only a minor portion of healthcare professionals with OUD (OIPs) abuse their prescription authority to support personal drug habits, this would amount to a serious problem.

A 2020 study confirmed OUD as the most common substance use disorder among anesthesiologists. The study examined two sources of data: death certificates and substance abuse cases reported to the American Board of Anesthesiology for physicians who completed an anesthesiology residency between 1977 and 2013. The rate of substance use disorders among these physicians was higher than that of the general population. Death certificates indicated a substance use disorder was the cause of death for 18% of the anesthesiologists. Among cases reported to the board, OUD was the most common problem, with 47% abusing opioids intravenously and 17 abusing opioids orally.^19^

An investigation of over 200 state and federal drug enforcement cases brought against healthcare providers between 2009 and 2014 found that at least 15% involved “practitioners stealing drugs for personal use.”^20^ Similarly, a review of 100 state licensing board cases of egregious ethical violations occurring in 28 states between 2008 and 2013 found that 17% were motivated by the doctors' own substance use disorders.^21^ Of these cases, 93% involved opioid prescriptions. Over 97% involved solo or small group practitioners.^22^

Research has not yet attempted to quantify the extent to which OIPs contribute to the ongoing epidemic. However, the above findings suggest OIPs represent a significant contributing factor in the nation's ongoing opioid crisis. The conservative assumption that the rate of OUD among providers is the same as it is for the general population (4.1%), there would be approximately 53 000 OIPs with prescriptions privileges. If even 20% of this subset of OIPs abuse their prescription privileges to support their own addictions, this unethical and unsafe practice would include over 10 000 OIPs or about 0.001% of the 10 million American adults suffering from OUD. Reviewing the impact of substance use disorders and diversion among physicians, Bryson noted that “diversion of medications for personal recreational use and for sale to those who have become addicted to these mind‐altering chemicals is nothing new, and history tells us that physicians were among the first to experiment with alternative uses for many of these agents.”^23^

With respect to the opioid epidemic, veterinarians warrant a callout as part of the healthcare community. While veterinarians neither prescribe nor dispense opioids to humans, they have opioid prescription authority and usually keep stocks of opioids within their clinics. They receive training on how to recognize attempts by animal owners to obtain opioids for human use. Advice from the FDA to veterinarians regarding opioids is refreshingly specific about addiction (eg, mentioning how to recognize addiction among employees). It also offers pragmatic considerations such as, “States such as Colorado and Maine require veterinarians to look at a pet owner's past medication history before dispensing opioids or writing an opioid prescription.” However, even this guide avoids the topic of addiction among veterinarians themselves.^24^

### 2.2 Provider monitoring programs

Almost every state has a program that coordinates confidential, therapeutic, and nonpunitive intervention for doctors and other healthcare professionals with a history of substance abuse. These provider monitoring programs stem from a 1973 AMA Council on Mental Health report that “recognized the significant scope of problems affecting physicians, the failure of physicians to seek help, and the ‘conspiracy of silence’ surrounding alcoholism and drug dependence.”^25^ The landmark report “helped to reorient physician impairment from a disciplinary issue to an illness requiring rehabilitation.”^26^

These programs are predicated on the view that it is possible to suffer from the problem of addiction without the condition causing permanent impairment. Namely, people can “recover” or go into “remission.” The programs coordinate assessment, treatment, and treatment compliance monitoring services. They offer a “collaborative process that leads to restored lives for the affected physician as well as patient safety.”^26^ Whenever possible, they avoid bringing participants to the attention of either medical boards or law enforcement agencies. Although the Federation of State Physician Health Programs (FSPHP) that advocates for use of these monitoring programs includes the word “physician” in the its title, most programs also serve dentists and allied health professionals.

An anonymous survey of physicians who had been referred to a state monitoring program indicated over 90% would recommend the program to others.^27^ Another study involving over 800 physicians who participated in such programs in 16 different states found that over 80% successfully completed the program. During the monitoring phase, 19% tested positive for drug or alcohol use. However, among program completers who returned to practice under monitoring conditions, almost 80% were still in practice 5 years later. Another 11% had had their licenses revoked, 3 % had retired, 3 % had died, and 3 % were of unknown status. Such findings suggest provider monitoring programs “provide an appropriate combination of treatment, support, and sanctions to manage addiction among physicians effectively,” and yield markedly better outcomes than observed among the general population.^28^ With evidence of high satisfaction and success among participants, particularly among those with substance use disorders, linkages have grown even stronger between monitoring programs and licensing boards. To be most effective, however, such programs must operate independently of medical and healthcare licensing boards regardless of whether they receive any funding from these boards and/or related professional societies. Indeed, FSPHP has taken strong steps to guard against conflicts of interest related to funding sources.^29^

### 2.3 Healthcare's hidden curriculum

Though ethical credos and standards of professionalism direct physicians to protect patients from impaired colleagues, a 2010 survey of 2038 physicians reported that almost a third of the 17% of responders with knowledge of an impaired colleague did not report that colleague to relevant authorities.^6^ Over a third of all respondents in this survey did not agree that physicians should report impaired colleagues at all, citing fear of retribution, belief that someone else would or should report, or that either no action or excessive punishment would result.^6^ This gap between what physicians know and how they actually behave may be attributed to the hidden curriculum, an unintended learning and socialization process in professional training that strongly influences beliefs, attitudes and behaviors.^26^, ^27^ For example, though the sanctioned formal medical school curriculum includes learning and skills objectives focused on empathic and patient‐centered care, student and resident “apprentices” may simultaneously experience negative attitudes toward or disparaging comments about patients with substance use disorders from their “master” role‐model clinician teachers. The hidden curriculum infiltrates every formal and informal arena of medical training, the hierarchal organizational structure of medical training and both academic and administrative policies and often discourages open disagreement, questioning authority, or asking for help.^26^

Deliberately deconstructing the hidden curriculum is no mean task, though the American College of Physicians and several other authors offer well‐structured guidance.^13^, ^26^, ^27^, ^28^ In a root‐cause analysis of a critical event involving the death of a medical student affected by OUD, Lucey et al, offer structural interventions during medical training for closing the gap between evidence‐based and actual physician behavior related to OIPs. These include normalizing confidential disclosure of substance use disorder upon matriculation; creating specific curricula around substance use disorder in health care professionals; and providing monitoring, treatment support and academic accommodations for affected students toward effective remission and successful licensing.^13^ Given Lucey et al estimated that by 2019, as many as 1900 United States medical students were suffering from OUD, this problem could affect the medical profession for years to come. Medical education, including continuing medical education, must change if we hope to optimize help for OIPs.

### 2.4 Systematically overcoming denial in healthcare

Comparable to healthcare's history of once having been in denial about the prevalence of medical errors, the profession has yet to fully and publicly acknowledge the apparently sizeable number of OIPs or their contribution to the opioid epidemic. For medical errors, things changed overnight in 1999 with the publication of the now seminal 312‐page monograph by the Institute of Medicine, aptly entitled *To Err is Human: Building a Safer Health System*.^30^ Release of the report “broke a long‐established wall of silence,” marking a turning point in medical history, and the start of the patient safety movement.^31^

The media's focus on the report and the federal government's response to it set critical actions in motion. The government set a goal of cutting medical errors in half over the next 5 years and Congress allocated $50 million for patient safety research. Equally important, the report removed the profession's own blinders to the magnitude of the problem. Until then, medical professionals had no way of gauging how isolated errors they witnessed or experienced added up on a clinic, hospital, community, or national level.^32^ Ever since, healthcare leaders, national and state organizations, and local hospitals have been taking systematic steps to reduce medical errors. Consequently, every United States hospital has a patient safety program in place, and virtually every healthcare worker is now familiar with some core patient safety terminology and prevention strategies.

Some patient safety programs have advanced to the point of requiring employed physicians to fully disclose medical errors to patients and families, one of the most challenging and potentially conflict‐ridden ethical obligations a provider will ever face. Notably, Ascension Health successfully increased its employee endorsement of a new full disclosure policy from 10% to 77% over a 27‐month period. A 221% increase in the rate of disclosures ensued across all of its 70 hospitals and hundreds of outpatient facilities.^33^ Ascension Health achieved this level of transformative improvement by establishing clear policy and an associated protocol containing highly scripted language (see Table 2).

**Table 2:** Overview of a patient safety initiative on the historically off‐limits topic of disclosing medical errors^a^

With federal funding, a large healthcare system piloted and refined a program to promote full disclosure—a commitment to communicating openly and honestly with patients and families about unexpected medical errors (a long‐established but often ignored ethical standard). Before final program rollout, teams of experts held meetings to introduce it in all local facilities. The program's protocol used highly scripted language to foster a high rates of provider compliance, consistency, and success	
The event	The scripted response
Potential medical error	“We are sorry that this event occurred and want you to know it is being reviewed carefully to determine the cause. As soon as this assessment is completed, we will meet with you to let you know the findings”
Error‐free adverse event	“We are very sorry that this event has occurred. We have completed the review and the event was not preventable for the following reasons”
Healthcare‐induced harm	“We are very sorry that our actions led to this very disappointing outcome. We would like to explain what happened and what changes we have made so this will not happen again. We will work with you to try to make you whole and earn back your trust”

a Ascension health's full disclosure protocol.^32^

Research has shown that the practice of full disclosure can substantially *decrease* the number of claims, lawsuits, and time from reporting to resolution as well as liability, compensation, and administrative costs.^34^ Full disclosure also guards against the costly second victim phenomenon “whereby health care workers are also traumatized by the same events that harm patients.”^35^ The success of this well‐coordinated effort to address one of healthcare's thorniest issues—an issue that providers have historically resisted addressing proactively—provides both inspiration and practical guidance for designing a framework and interventions to address the equally thorny and uncomfortable issue of helping OIPs.

### 2.5 Building and leveraging local capacity

One critical lesson learned from solving some of the world's most challenging healthcare problems is the concept that *overcoming deep‐rooted issues often has less to do with figuring out what people must do and more to do with figuring out how to get people to do what is necessary*.^32^, ^36^, ^37^ Indeed, the OIP component of the opioid crisis is unlikely to be solved until local communities develop specific and pragmatic guidance on how healthcare professionals should respond to an impaired colleague irrespective of that colleague's discipline or employing organization. Consider the fact that, in the United States, dentists are among the most frequent prescribers of opioids (second only to family physicians).^38^ Most dentists work in small or solo practices, which are the vulnerable circumstances under which egregious ethical violations seem prone to occur. Research has shown that compared to providers working in hospitals and clinics, those practicing in solo or small practices are equally likely to have direct knowledge of impaired colleagues, but about twice as unlikely to address the problem.^39^

Building community‐based networks (ie, coalitions) represents a proven and cost‐efficient way to meaningfully engage diverse stakeholders in creating workable, local‐level solutions to vexing healthcare issues.^32^ Rather than relying on individual organizations to solve seemingly intractable problems, coalitions pool community resources to better tackle the factors underlying complex problems. The resulting synergy makes it possible to accomplish goals that no single organization could achieve on its own.^40^ Such collaboration will be necessary to adequately tackle the opioid crisis but may not occur without encouragement and guidance from national and state agencies.

Because a local/regional medical center is usually the largest healthcare entity in any given community, it is most likely to have the appropriate staff with which to coordinate a network that represents all types of licensed providers. While not every impaired provider will be formally affiliated with their respective local/regional medical centers, these centers will presumably serve every impaired provider's patients.

## 3 CONCLUSION

The number of OIPs abusing their prescription privileges to support personal drug addictions is likely to comprise only a tiny fraction of the 10 million adults with OUD; however, that tiny fraction could amount to at least 10 000 OIPs engaging in unethical and unsafe opioid prescribing practices. This arguably conservative estimate equates to at least 200 providers per state, leaving few, if any, communities totally immune. The United States needs a national‐level healthcare policy that directs communities to develop and implement “how‐to” guidance to ensure healthcare professionals can effectively meet their ethical obligation to encourage the rehabilitation of OIPs without compromising patient safety or requiring criminal prosecution. Such guidance must be customizable to local needs and capacities. It should include clear action steps healthcare professionals can employ regardless of their professional disciplines or employing organizations and specific strategies for connecting with state‐level provider monitoring programs. As the nation reflects on longstanding racial biases within the law enforcement community, the health profession must also face aspects of its own hidden curriculum. Meanwhile, healthcare leaders should continue to support the DOJ initiative to prosecute all unimpaired providers who willfully abuse their prescription privileges. Both healthcare and law enforcement strategies are necessary to most effectively combat the societal‐level opioid crisis.

## CONFLICT OF INTEREST

The authors declare no conflicts of interest.

## AUTHOR CONTRIBUTIONS

Conceptualization: Gretchen LeFever Watson, Nancy J. Selfridge, Bruce E. Wright

Investigation: Gretchen LeFever Watson, Nancy J. Selfridge, Bruce E. Wright

Project administration: Gretchen LeFever Watson

Supervision: Nancy J. Selfridge

Writing ‐ original draft preparation: Gretchen LeFever Watson, Nancy J. Selfridge, Bruce E. Wright

Writing ‐ review and editing: Gretchen LeFever Watson, Nancy J. Selfridge, Bruce E. Wright

 All authors have read and approved the final version of the manuscript.

## TRANSPARENCY STATEMENT

Gretchen LeFever Watson affirms that this manuscript is an honest, accurate, and transparent account of the study being reported; that no important aspects of the study have been omitted; and that any discrepancies from the study as planned (and, if relevant, registered) have been explained.

## Data Availability

Data sharing is not applicable to this article as no new data were created or analyzed in this study.

## References

[1] Centers for Disease Control and Prevention . Opioids Portal. America's Drug Overdose Epidemic: Data to Action; 2019 https://www.cdc.gov/opioids/index.html

[2] Federal health care fraud takedown in northeastern U.S. results in charges against 48 individuals [press release]. https://www.justice.gov/opa/pr/federal-health-care-fraud-takedown-northeastern-us-results-charges-against-48-individuals2019

[3] American Medical Association . AMA Code of Medical Ethics. Chicago, IL: American Medical Association; 2016.

[4] American Dental Association . Principles of Ethics & Code of Professional Conduct. Chicago, IL, ADA Council on Ethics, Bylaws and Judicial Affairs: American Dental Association; 2018.

[5] Sulmasy LS , Bledsoe TA , ACP Ethics, Professionalism and Human Rights Committee . American College of Physicians ethics manual: seventh edition. Ann Intern Med. 2019;170(2_Supplement):S1‐S32. 10.7326/M18-2160 30641552

[6] Candilis PJ , Kim DT , Sulmasy LS , ACP Ethics, Professionalism and Human Rights Committee . Physician impairment and rehabilitation: reintegration into medical practice while ensuring patient safety: a position paper from the American College of Physicians. Ann Intern Med. 2019;170(12):871‐879. 31158847 10.7326/M18-3605

[7] Greenhouse L . Justices rule police do not have a constitutional duty to protect someone. *The New York Times* 2005 https://www.nytimes.com/2005/03/22/politics/justices-hear-debate-on-whether-police-must-intervene.html. Accessed November 10, 2019.

[8] Daugherty S. A Virginia Beach dentist tried to cover up his bogus prescriptions. *The Virginian‐Pilot* October 2, 2019. https://www.pilotonline.com/news/crime/vp‐nw‐bogus‐prescriptions‐20191002‐dbk2mzqcuzhqtafcsbecjn6v34‐story.html. Accessed November 10, 2019.

[9] Dentist pleads guilty to running oxycodone conspiracy [press release]. United States Department of Justice; 2019.

[10] Virginia Board of Dentistry . Consent order for Gary A. Hartman. Virginia Board of Dentistry; December 20, 2018.

[11] Watson GBL . Health care professionals must help their own. *The Virginian‐Pilot* 2019 https://www.pilotonline.com/opinion/columns/vp-ed-column-watson-1103-20191103-dh5xxwhbtbanxbp56aqzocpcs4-story.html

[12] Merlo LJ , Teitelbaum SA , Thompson K . Substance use disorders in physicians: epidemiology, clinical manifestations, identification, and engagement. *UpToDate* 2019 (Literature current through November 2019). https://www.uptodate.com/contents/substance‐use‐disorders‐in‐physicians‐epidemiology‐clinical‐manifestations‐identification‐and‐engagement/print. Accessed December 9, 2019.

[13] Lucey CR , Jones L , Eastburn A . A lethal hidden curriculum – death of a medical student from opioid use disorder. N Engl J Med. 2019;381(9):793‐795. 31461592 10.1056/NEJMp1901537

[14] Hughes P , Brandenburg N , Baldwin D , et al. Prevalence of substance use among US physicians. Jama. 1992;11(268):18. 1348789

[15] Merlo L , Gold M . Prescription opioid abuse and dependence among physicians: hypotheses and treatment. Hav Rev Psychiatry. 2008;16(3):181‐194. 10.1080/10673220802160316 18569039

[16] Nance E , Davis C , Huner J . A comparison of male and female physicians treated for substance use and psychiatric disorders. Am J Addict. 1995;4:156‐162.

[17] National Institutes of Health . Rates of nonmedical prescription opioid use and opioid use disorder double in 10 years [press release]; June 2, 2016. https://www.niaaa.nih.gov/news‐events/news‐releases/rates‐nonmedical‐prescription‐opioid‐use‐and‐opioid‐use‐disorder‐double‐10

[18] Saha TD , Kerridge BT , Grant B . Nonmedical prescription opioid use and DSM‐5 nonmedical prescription opioid use disorder in the United States. J Clin Psychiatry. 2016;77(6):772‐780. 27337416 10.4088/JCP.15m10386 PMC5555044

[19] Warner DO , Berge K , Sun H , Harman A , Wang T . Substance use disorder in physicians after completion of training in anesthesiology in the United States from 1977 to 2013. Anesthesiology. 2020;133:342–349. 32282430 10.1097/ALN.0000000000003310

[20] Eisler P. Doctors, medical staff on drugs put patients at risk. *USA Today*. April 15, 2014.

[21] DuBois JM , Anderson EA , Chibnall JT , et al. Preventing egregious ethical violations in medical practice: evidence‐informed recommendations from a multidisciplinary working group. J Med Regul. 2018;104(4):23‐31. 30984914 10.30770/2572-1852-104.4.23 PMC6461379

[22] DuBois JM , Chibnall JT , Anderson EE , Eggers M , Baldwin K , Vasher M . A mixed‐method analysis of reports on 100 cases of improper prescribing of controlled substances. J Drug Issues. 2016;46(4):457‐472. 28663601 10.1177/0022042616661836 PMC5485258

[23] Bryson EO . The impact of chemical dependency on health care professionals involved with the delivery of anethsesia. Int Anesthesiol Clin. 2020;58(1):45‐49. 31800415 10.1097/AIA.0000000000000257

[24] Food and Drug Administration . The opioid epidemic: what veterinarians need to know; 2018. https://www.fda.gov/animal-veterinary/resources-you/opioid-epidemic-what-veterinarians-need-know

[25] American Medical Association . The sick physician. Impairment by psychiatric disorders, including alcoholism and drug dependence. Jama. 1973;223:684‐687. 4739202

[26] Angres DH , John A , Bettinardi‐Angres K , Agarwal G . The forensic evaluation and rehabilitation of the impaired physician. Psychiatr Ann. 2019;49(11):487‐491.

[27] Merlo LJ , Greene WM . Physician views regarding substance use‐related participation in a state physician health program. Am J Addict. 2010;19(6):529‐533. 20958849 10.1111/j.1521-0391.2010.00088.x PMC2959195

[28] McLellan TA , Skipper GE , Campbell M , Dupont RL . Five year outcomes in a cohort study of physicians treated for substance use disorders in the United States. BMJ. 2008;337(a2038):1‐6. 10.1136/bmj.a2038 PMC2590904 18984632

[29] Federation of State Physician Health Programs . *AMA Model Bill: Physician Health Programs Act*; 2018. https://www.fsphp.org/ama-model-bill-physician-health-programs-act. Accessed November 21, 2019.

[30] Kohn L , Corrigan J , Donaldson M . To Err Is Human: Building a Safer Health System. Washington, DC: Institute of Medicine; 1999. 25077248

[31] Watson G . The hospital safety crisis. Society. 2016;53(4):339‐347.

[32] Watson GL . Your Patient Safety Guide: How to Protect Yourself and Others from Medical Errors. Lantham, MD: Rowman & Littlefield; 2019.

[33] Hendrich A , McCoy CK , Gale J , Sparkman L , Santos P . Ascension Health's demonstration of full disclosure protocol for unexpected events during labor and delivery shows promise. Health Aff (Millwood). 2014;33(1):39‐45. 24395933 10.1377/hlthaff.2013.1009

[34] Kachalia A , Little A , Isavoran M , Crider L‐M , Smith J . Greatest impact of safe harbor rule may be to improve patient safety, not reduce liability claims paid by physicians. Health Aff (Millwood). 2014;33(1):59‐66. 24395936 10.1377/hlthaff.2013.0834

[35] Wu AW , Boyle DJ , Wallace G , Mazor KM . Disclosure of adverse events in the United States and Canada: an update, and a proposed framework for improvement. J Public Health Res. 2013;2(3):e32. 25170503 10.4081/jphr.2013.e32 PMC4147741

[36] Pascale R , Sternin J , Sternin M . The Power of Positive Deviance: How Unlikely Innovators Solve the World's Toughest Problems. Boston, MA: Harvard Business Press; 2010.

[37] Gandhi TK , Kaplan GS , Leape L , et al. Transforming concepts in patient safety: a progress report. BMJ Qual Saf. 2018;27(12):1019‐1026. 10.1136/bmjqs-2017-007756 PMC6288701 30018115

[38] Suda KJ , Durkin MJ , Calip GS , et al. Comparison of opioid prescribing by dentists in the United States and England. Jama. 2019;2(5):1‐8. 10.1001/jamanetworkopen.2019.4303 PMC6632141 31125102

[39] DesRoches C , Rao S , Fromson J , et al. Physicians' perceptions, preparedness for reporting, and experiences related to impaired and incompetent colleagues. Jama. 2010;14(30):187‐193. 10.1001/jama.2010.921 20628132

[40] Foster‐Fishman P , Berkowitz S , Lounsbury D , Jacobson S , Allen N . Building collaborative capacity in community coalitions: a review and integrative framework. Am J Community Psychol. 2001;29:241‐261. 11446279 10.1023/A:1010378613583

